# Breaking Barriers: Evaluating Challenges in Advancing Periodontal Ligament Cell-Derived Organoids

**DOI:** 10.3390/dj13090422

**Published:** 2025-09-13

**Authors:** Luiza de Oliveira Matos, Mariane Beatriz Sordi, Anahid Ahmadi Birjandi, Paul Thomas Sharpe, Ariadne Cristiane Cabral Cruz

**Affiliations:** 1 Centre for Craniofacial and Regenerative Biology, Faculty of Dentistry, Oral & Craniofacial Sciences, Guy’s Hospital, King’s College London, London SE1 9RT, UK; matosluizaa@gmail.com (L.d.O.M.); marianesordi@hotmail.com (M.B.S.); anahid.ahmadi_birjandi@kcl.ac.uk (A.A.B.); paul.sharpe@kcl.ac.uk (P.T.S.); 2 Centre for Dental Implants Research, Federal University of Santa Catarina, Florianópolis 88040-900, SC, Brazil; 3 Applied Virology Laboratory, Federal University of Santa Catarina, Florianópolis 88040-900, SC, Brazil

**Keywords:** periodontal ligament cells, organoids, three-dimensional cell culture, periodontal regeneration

## Abstract

The objective of this review was to critically evaluate the available literature on the development of periodontal ligament organoids. Articles concerning periodontal ligament organoids were considered eligible. References were selected in a two-phased process. Electronic databases PubMed and Scopus were screened up to June 2024, yielding 1101 studies. After removing duplicates, titles, and abstracts were screened, resulting in 44 articles being included in this review. A detailed analysis of the included articles was organized into four categories: (1) the cell lineages used, including the simultaneous use of two or more cell types, (2) the extracellular matrix composition, (3) the organoid preparation methods, and (4) the characterization techniques employed. The main findings show that collagen combined with biodegradable polymers—such as poly(caprolactone), poly(glycolic acid), and poly(lactic acid)—is the most used material. Periodontal ligament cells and periodontal fibroblasts were the most used cell types, due to their role in extracellular matrix remodeling. The most frequent analyses performed included alkaline phosphatase, extracellular matrix mineralization, and gene expression, providing insights into differentiation and periodontal regeneration. Cementogenic differentiation was the most studied, followed by osteogenic, chondrogenic, adipogenic, and epithelial differentiation. However, challenges remain, including methodological inconsistencies and the need for scaffold optimization. Future research should focus on standardizing protocols, improving biomaterials, and integrating bioprinting techniques to improve clinical translation.

## 1. Introduction

Cell culture is a fundamental technique that allows for the controlled study of cell behavior, function, and responses to different stimuli under well-defined conditions [1]. For a long time, two-dimensional (2D) cell culture served as the benchmark in experimental studies, involving the seeding of cells onto rigid plastic plates to form a monolayer [2,3]. These 2D techniques have been widely applied in medicine and biotechnology, contributing to advancements in drug development, tissue engineering, and disease modeling [4]. However, it has become evident that 2D cultures fail to accurately replicate the physiological conditions within a living organism [5]. As a result, these systems can lead to diminished cellular replication capacity, altered colony formation, and impaired differentiation [1].

In contrast, cells in the human body exist within a three-dimensional (3D) environment, supported by the surrounding extracellular matrix (ECM). In this environment, cells receive nutrients, oxygen, and growth factors through diffusion gradients and the permeation properties of the ECM [6,7]. A defining feature of 3D cell culture systems is their ability to support cell-to-cell and cell-to-ECM interactions, along with paracrine signaling through the diffusion of cell-secreted factors [1,8]. With advances in biotechnology, 3D cell culture models have emerged as more physiologically relevant platforms for studying human physiology and disease. Among these, spheroids and organoids are two widely used models.

Spheroids are simple 3D aggregates of cells, while organoids are more complex structures capable of self-renewal, differentiation, and organization into spatial arrangements that closely mimic human organs [9]. Organoids exhibited the self-organization of organ-specific cell types into in vivo-like structures and can recapitulate some organ functions. These models are typically formed by combining cells, ECM components, and growth factors, and they have demonstrated great potential in drug screening, human disease modeling, and personalized medicine [10,11].

Despite the advantages of 3D organoid technology over traditional 2D cultures and animal models, this field is still in its early stages, with several challenges yet to be addressed. Organoid systems for tissues such as the intestine [12,13], liver [14], and lungs [15] have shown notable progress. Tooth organoids have emerged, successfully reproducing epithelial–mesenchymal interactions and generating enamel-, dentin-, and root-like structures, opening new avenues for dental tissue research [16,17,18]. In parallel, the development of periodontal ligament organoids is still in its nascent stage and requires further methodological refinement.

In this review, we propose to critically evaluate the available in vitro and in vivo literature concerning periodontal ligament organoids and identify the challenges that must be addressed to advance this 3D cell culture model. Our goal is to provide valuable insights into the potential application of periodontal ligament organoids in regenerative tissue engineering and personalized periodontal treatments, while highlighting the barrier to their clinical translation.

## 2. Methods

An electronic search of the PubMed and Scopus databases was conducted up to June 2024. The following search terms were employed: (periodontal ligament cells OR PDL OR periodontal ligament stem cells OR dental pulp mesenchymal stem cells OR dental follicle progenitor cells OR stem cells from apical papilla OR oral fibroblasts OR gingival fibroblasts OR oral epithelial cells OR gingival epithelial cells) AND (organoid OR three-dimensional culture). The search was limited to publications in the English language and included in vitro, in vivo, and clinical studies, without restricting the year of publication.

The following exclusion criteria were considered: studies on cell culture that did not evaluate organoids, case reports, protocols, short communications, personal opinions, letters, posters, book chapters, conference abstracts, full-text not available, duplicate data (e.g., dissertations or theses with corresponding published articles), literature reviews, and systematic reviews.

A two-phase selection process was performed. In phase 1, the title and abstract were screened to identify potentially eligible studies. Phase 2 involved a full-text reading of eligible articles. Subsequently, a narrative review was conducted to discuss the results, focusing on cell lineages and origin, the extracellular matrix components employed, and the main findings.

## 3. Results

### 3.1. Study Selection

**Figure 1:**
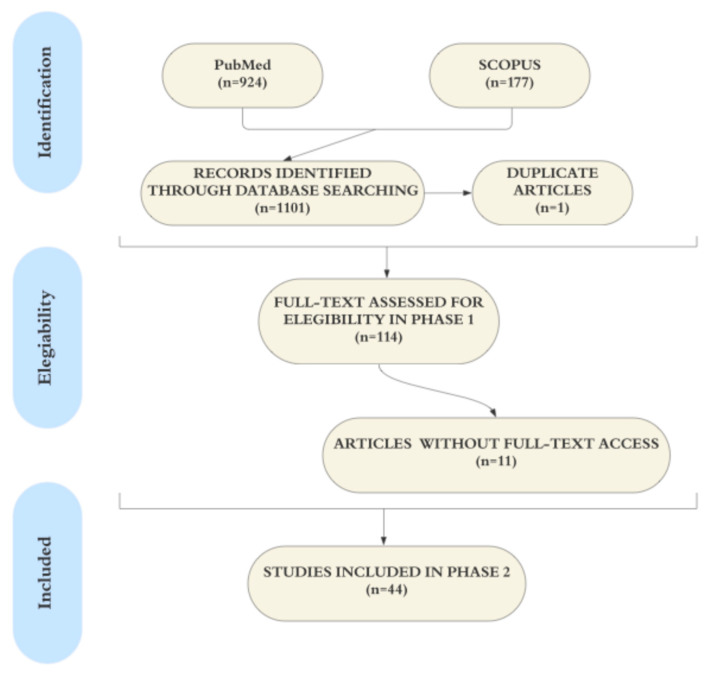
Flowchart of the literature search and selection of the included studies. References were selected in a two-phase process. Electronic databases (PubMed and SCOPUS) were searched up to June 2024.

As depicted in Figure 1, the initial search yielded 924 studies from PubMed (June 1967 to June 2024) and 177 studies from Scopus, totaling 1101 articles. After removing one duplicate, the titles and abstracts of the remaining 1100 articles were screened (Phase 1), resulting in 114 studies. Due to a lack of full-text access, 13 publications were excluded, leaving 103 articles. No clinical studies were identified. After full-text review (Phase 2), 44 articles were included in this review.

### 3.2. Cells

**Figure 2:**
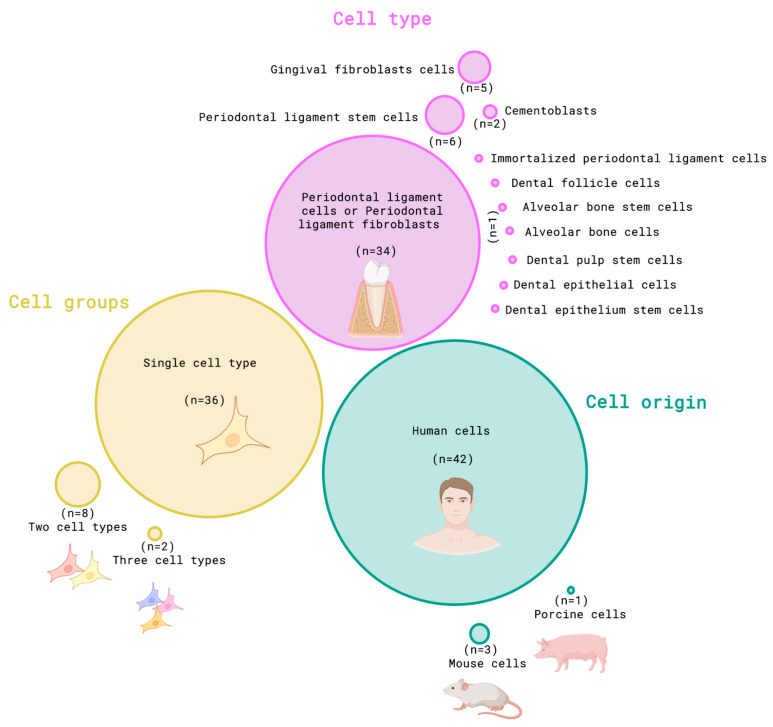
Venn diagram concerning the cell types, origin, and groups employed for organoid construction. Cell lineages employed for organoid construction included alveolar bone cells (*n* = 1), alveolar bone stem cells (*n* = 1), dental pulp stem cells (*n* = 1), dental epithelium cells (*n* = 1), dental epithelium stem cells (*n* = 1), cementoblasts (*n* = 2), dental follicle cells (*n* = 2), immortalized periodontal ligament cells (*n* = 2), gingival fibroblasts (*n* = 5), periodontal ligament stem cells (*n* = 6), and periodontal ligament cells or periodontal ligament fibroblast (*n* = 34). Most included studies used one cell type (*n* = 36), followed by studies using two types (*n* = 8) and three types (*n* = 3). Human cells (*n* = 42) were used in most included studies, followed by cells from mice (*n* = 3) and from porcine (*n* = 1).

As illustrated in Figure 2, most studies (*n* = 34) investigated periodontal ligament cells or periodontal ligament fibroblasts [5,16,17,18,19,20,21,22,23,24,25,26,27,28,29,30,31,32,33,34,35,36,37,38,39,40,41,42,43,44,45,46,47]. Periodontal ligament stem cells (*n* = 6) [48,49,50,51,52,53] and gingival fibroblasts (*n* = 5) [23,48,54,55,56] were also studies. Other cell types included immortalized periodontal ligament cells (*n* = 2) [57,58], dental follicle cells (*n* = 2) [58,59], cementoblasts (*n* = 2) [42,59], dental epithelium stem cells (*n* = 1) [9], dental epithelial cells (*n* = 1) [35], dental pulp stem cells (*n* = 1) [53], alveolar bone stem cells (*n* = 1) [53], and alveolar bone cells (*n* = 1) [60].

Most studies evaluated a single cell type (*n* = 36) [5,9,16,17,18,19,21,22,23,24,25,26,27,28,29,30,31,34,35,36,37,38,40,41,42,43,44,47,48,49,51,52,53,60,61], while others included two (*n* = 8) [23,35,42,48,54,58,60,62] or three cell types (*n* = 2) [53,59]. Most articles employed human cells (*n* = 42) [5,16,17,18,19,20,21,22,23,24,25,26,27,28,29,30,31,33,34,35,37,38,39,40,41,42,43,44,46,47,48,49,50,51,52,53,55,56,59,60,61], with fewer using animal cells, including mouse (*n* = 3) [9,39,59] and porcine (*n* = 1) [35]. Details regarding cell types and methodology are provided in Table 1, Table 2 and Table 3.

### 3.3. Extracellular Matrix Component

**Figure 3:**
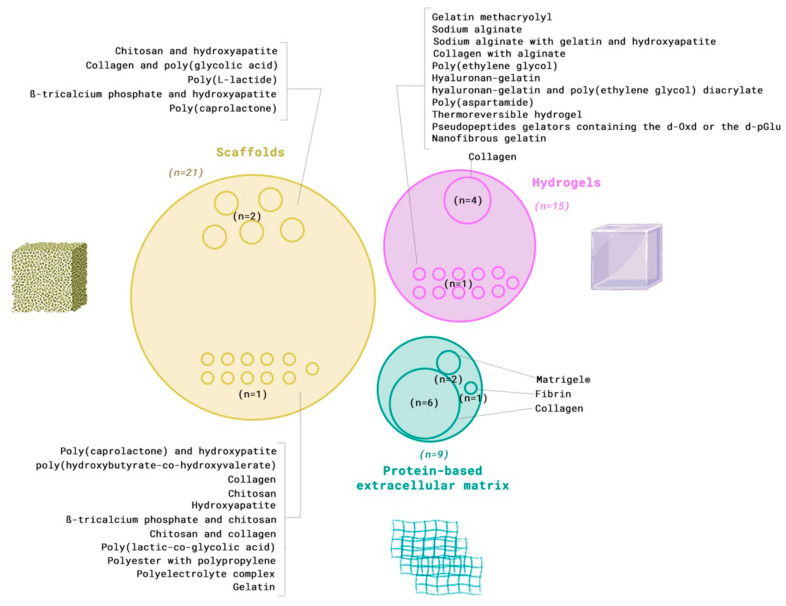
Venn diagram demonstrating the extracellular matrix component used for organoid fabrication.

Organoid fabrication involved various extracellular matrix (ECM) types, classified as solid scaffolds, hydrogels, or protein-based extracellular matrix, with both synthetic and natural origins. As presented in Figure 3 and Table 1, solid scaffolds were the most frequently used biomaterial (*n* = 21) [22,24,26,30,32,33,37,40,41,42,43,44,45,47,48,52,53,56,57,59,61], followed by hydrogels (*n* = 13) [5,19,20,25,27,28,29,31,36,39,50,51,55] and protein-based extracellular matrix (*n* = 9) [9,21,23,34,35,47,54,60,62]. 

Materials included poly(caprolactone) (*n* = 2) [22,26], ß-tricalcium phosphate associated with hydroxyapatite (*n* = 2) [33,52], poly(L-lactide acid) (*n* = 2) [32,37], collagen associated with poly(glycolic acid) (*n* = 2) [41,47], poly(caprolactone) with hydroxyapatite (*n* = 1) [53], poly(hydroxybutyrate-co-hydroxyvalerate) (*n* = 1) [48], gelatin (*n* = 1) [49], titanium and hydroxyapatite core (*n* = 1) [24], collagen (*n* = 1) [57], chitosan (*n* = 1) [30], hydroxyapatite (*n* = 1) [40], poly(lactic-co-glycolic acid) (*n* = 1) [59], polyester with polypropylene (*n* = 1) [56], and polyelectrolyte complex (*n* = 1) [61]. Most scaffolds were polymer-based, reflecting their versatility and established use in tissue engineering. Within this group, natural polymers and composites incorporating ceramics showed superior cell adhesion [30,41,47,53,57], proliferation [30,33,41,47,52,53], and differentiation [33,41,47,52,53]. In contrast, purely synthetic polymers exhibited limited biological performance unless modified or combined with bioactive components [32,37,41,47,59]. Consequently, polymers remain the predominant class of scaffolds, but natural-derived and composite types demonstrated greater regenerative potential.

Hydrogel were used in 15 studies (*n* = 15) [5,9,19,20,25,27,28,29,31,36,39,49,50,51,55], as shown in Figure 3 and Table 2. The materials used for hydrogel preparation include gelatin methacryolyl (*n* = 1) [19], sodium alginate (*n* = 1) [19], sodium alginate with gelatin and hydroxyapatite (*n* = 1) [51], collagen (*n* = 4) [20,36,39,50], collagen with alginate (*n* = 1) [25], poly(ethylene glycol) (*n* = 1) [5], hyaluronangelatin (*n* = 1) [28], hyaluronangelatin and poly(ethylene glycol) diacrylate (*n* = 1) [27], poly(aspartamide) (*n* = 1) [29], thermoreversible hydrogel (*n* = 1) [31], Matrigel (*n* = 2) [9], nanofibrous gelatin (*n* = 1) [49], and pseudopeptides gelators containing the d-Oxd or the d-pGlu (*n* = 1) [55]. Hydrogels provide a 3D matrix suitable for cell culture, offering structural support and influencing cell behavior and tissue regeneration.

As depictable in Figure 3 and Table 3, protein-based extracellular matrices were reported in studies [21,23,34,35,46,54,60,62], with Matrigel® (*n* = 2) [9,62], collagen (*n* = 6) [21,34,35,46,54,60], and fibrin (*n* = 1) [23] being most used. These matrices play a crucial role in creating 3D microenvironments that mimic native conditions.

### 3.4. Organoids Preparation

**Figure 4:**
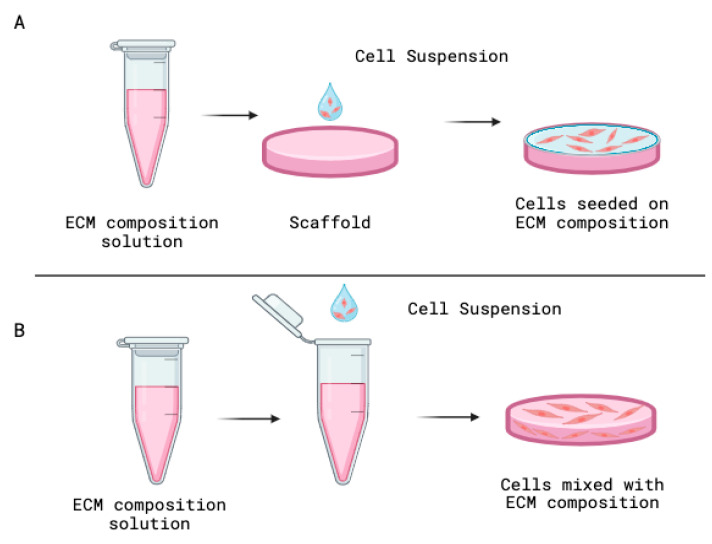
Methods used for organoid preparation. (**A**) A solid scaffold is made by extracellular matrix composition solution, and the cell suspension is seeded on the top of the scaffold. Introduce the cell suspension inside the extracellular matrix (ECM) composition solution and mix to development the organoid. (**B**) Introduce the cell suspension inside the extracellular matrix (ECM) composition solution and mix to development the organoid.

Two primary methods of organoid preparation were found in this literature review. In most articles (*n* = 34) [5,9,21,22,24,26,27,29,30,32,33,34,35,37,40,41,42,43,44,45,46,47,48,49,50,51,52,53,54,55,56,57,59,61] cells were directly onto a pre-formed scaffold made of extracellular matrix components (Figure 4A). In contrast, the second method (*n* = 9) [19,20,23,25,28,31,36,39,62] involves combining a cell suspension—composed of cells suspended in a cell culture medium—with a solution containing an extracellular matrix composite to facilitate organoid development (Figure 4B). Notably, the development of organoids using both methods was described once (*n* = 1) [60].

### 3.5. Characterization Methods

**Figure 5:**
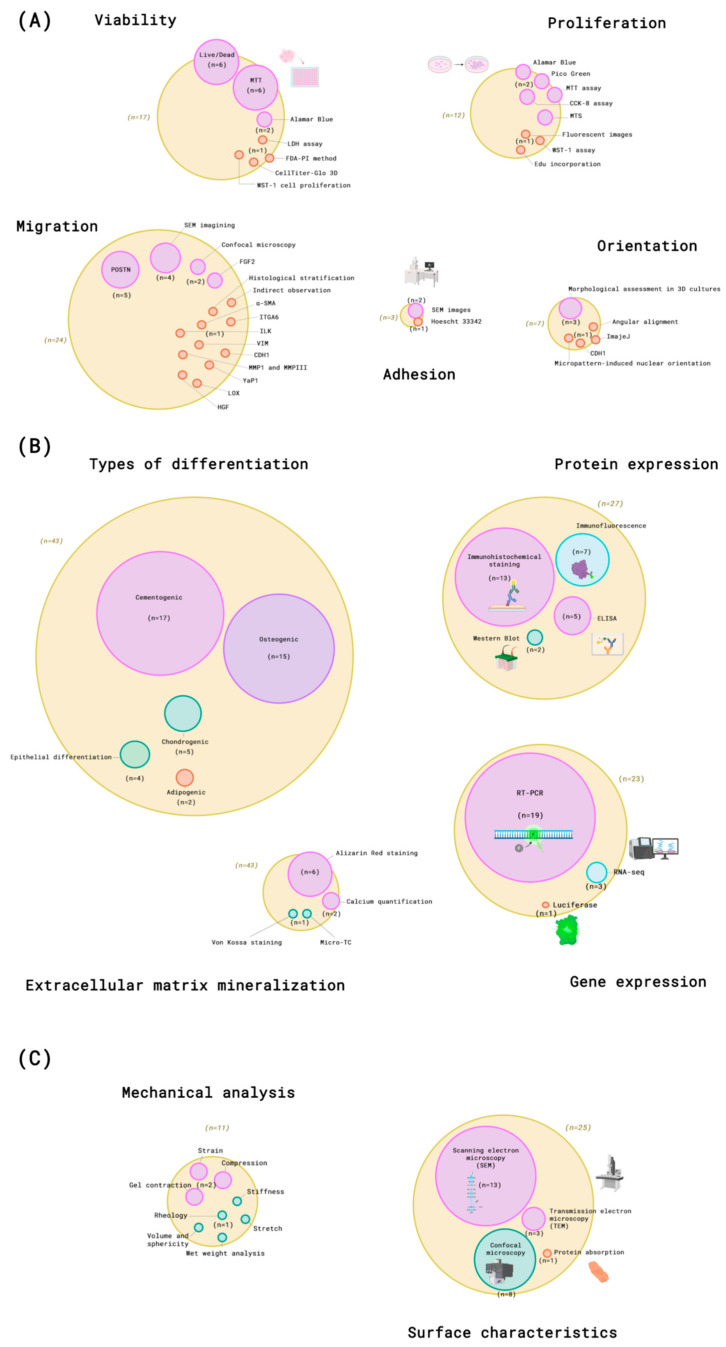
Venn diagram demonstrating findings concerning (**A**) cellular viability, proliferation, adhesion, migration, orientation, and (**B**) differentiation. (**C**) Organoid surface and mechanical characteristics were also evaluated. Legend: (**A**) CCK-8 assay—Cell Counting Kit-8; EdU incorporation—thymidine analog (5-ethynyl-2′-deoxyuridine) incorporation; FDA-P1 method—fluorescein diacetate; LDH assay—lactate dehydrogenase assay; MTS ([3-(4,5-dimethylthiazol-2-yl)-5-(3-carboxymethoxyphenyl)-2-(4-sulfophenyl)-2H-tetrazolium); MTT—(3-(4,5-dimethylthiazol-2-yl)-2,5-diphenyltetrazolium bromide); TSO SPS microscope—Triple Staining Observation with Structured Pattern Scanning; WST-1—Water-Soluble Tetrazolium Salt-1. (**B**) ELISA—enzyme-linked immunosorbent assay; RT-PCR—Real-Time Polymerase Chain Reaction; RNA-seq—RNA sequencing.

As depicted in Figure 5, to evaluate the organoids, some characteristics were analyzed, including cellular viability, adhesion, proliferation, migration, and differentiation. The surface characteristics and mechanical behavior of the organoids were also evaluated, along with inflammatory markers.

#### 3.5.1. Viability

Cellular viability determination (*n* = 17) [19,20,27,28,29,30,31,34,36,41,42,43,44,45,51,55,57] most performed were MTT (3-(4,5-dimethylthiazol-2-yl)-2,5-diphenyltetrazolium bromide) assay (*n* = 6) [31,34,42,43,44,45], followed by Live/Dead assay (*n* = 6) [19,20,30,36,51,57], Alamar Blue (*n* = 2) [19,41], lactate dehydrogenase (LDH) assay (*n* = 1) [55], fluorescein diacetate (FDA)–propidium iodide (PI) method (*n* = 1) [28], CellTiter-Glo 3D cell viability assay (*n* = 1) [27], and WST-1 cell proliferation reagent (*n* = 1) [29].

#### 3.5.2. Proliferation

Cellular proliferation (*n* = 12) [5,9,19,28,33,36,37,39,40,48,49,51] analysis included Alamar Blue (*n* = 2) [5,19], Pico Green (*n* = 2) [19,36], cell counting from fluorescent images (*n* = 1) [19], MTT assay (*n* = 2) [48,49], CCK-8 assay (*n* = 2) [33,51], MTS (3-(4,5-dimethylthiazol-2-yl)-5-(3-carboxymethoxyphenyl)-2-(4-sulfophenyl)-2H-tetrazolium) (*n* = 2) [28,37], WST-1 assay (*n* = 1) [40], and EdU incorporation (*n* = 1) [9].

#### 3.5.3. Adhesion

Cell adhesion (*n* = 3) [31,49,51] was evaluated by SEM images (*n* = 2) [31,51] and Hoechst 33342 (*n* = 1) [49].

#### 3.5.4. Migration

To assess cell migration, studies employed methods such as SEM imaging (*n* = 4) [21,36,41,45], confocal microscopy (*n* = 2) [49,50], histological stratification (*n* = 1) [60], and indirect observation of cell escape from hydrogels (*n* = 1) [19]. To analyze cell migration the following marks were used: alpha-smooth muscle actin (α-SMA) (*n* = 1) [20], Integrin Subunit Alpha 6 (ITGA6) (*n* = 1) [9], integrin-linked kinase (ILK) (*n* = 1) [25], Vimentin (VIM) (*n* = 1) [52], E-cadherin (CDH1) (*n* = 1) [57], matrix metalloproteinase 1 and 3 (MMP1 and MMPIII) (*n* = 1) [28], Yes-Associated Protein 1 (YaP1) (*n* = 1) [49], Lysyl Oxidase (LOX) (*n* = 1) [27], fibroblast growth factor 2 (FGF2) (*n* = 2) [32,37], Hepatocyte Growth Factor (HGF) (*n* = 1) [24], and periostin (POSTN) (*n* = 5) [5,21,41,49,57].

#### 3.5.5. Orientation

Cell orientation was evaluated through directionality analysis with ImageJ (National Institute of Health, version 1.53 t) (*n* = 1) [20], angular alignment under mechanical strain (*n* = 1) [39], micropattern-induced nuclear orientation (*n* = 1) [26], morphological assessment in 3D cultures (*n* = 3) [28,30,45], and E-cadherin (CDH1) (*n* = 1) [57].

#### 3.5.6. Differentiation

Concerning the differentiation, cementogenic (*n* = 17) [5,24,27,28,32,33,34,37,39,41,43,45,49,50,53,56,59] was the most evaluated, followed by osteogenic (*n* = 15) [5,24,28,32,33,34,37,39,41,43,45,49,53,56,59], chondrogenic (*n* = 5) [9,28,30,32,37], adipogenic (*n* = 2) [27,28], and epithelial differentiation (*n* = 4) [9,28,30,57]. The genes and proteins evaluated in cellular differentiation are listed in Table 4. To assess gene expression, real-time polymerase chain reaction (RT-PCR) (*n* = 19) [5,9,24,25,27,28,30,33,34,37,39,43,45,49,53,57,58,59,62] and RNA sequencing (RNA-seq) analysis (*n* = 3) [9,32,49] were utilized. For functional and cellular activity analysis, the Luciferase reporter gene assay (*n* = 1) [44] was used. Regarding protein expression, immunohistochemical staining (*n* = 13) [9,21,23,24,35,41,43,47,52,53,54,59,62], (*n* = 7) [23,24,25,26,34,49,52], enzyme-linked immunosorbent assay (ELISA) (*n* = 5) [23,28,44,46,56], Western blot (*n* = 2) [25,50], and Multiplex immunoassay (*n* = 1) [21] were employed.

Also, differentiation process was evaluated through alkaline phosphatase (ALP) (*n* = 16) [5,20,22,29,30,31,33,34,37,42,43,45,46,50,51,56] and extracellular matrix mineralization (*n* = 10) [5,24,33,34,42,49,50,58,59,61] determined by Alizarin red staining (*n* = 6) [33,34,49,50,58,61], Von Kossa staining (*n* = 1) [42], calcium quantification (*n* = 2) [5,50], and micro-CT (*n* = 1) [52].

Additionally, Hoechst-33358 staining (*n* = 1) [44] was used for DNA quantification. Colony-forming assays (*n* = 2) [30,62] were performed to evaluate cellular survival and clonogenic potential. Picrosirius red staining (*n* = 4) [24,36,37,49] and Masson’s trichrome (*n* = 2) [24,52] were used to assess collagen deposition and fibrosis. Hematoxylin and eosin (H&E) (*n* = 6) [24,43,44,45,56,59] and hemalum staining (*n* = 1) [56] were employed for general tissue morphology and cellular structure visualization. Titration assay was employed for viral detection (*n* = 1) [62] and inflammatory biomarkers were also evaluated (*n* = 1) [22].

#### 3.5.7. Surface Characteristics

The surface characterization analysis included scanning electron microscopy (SEM) (*n* = 13) [21,31,32,33,37,40,41,43,44,45,47,56,61], transmission electron microscopy (TEM) (*n* = 3) [9,21,56], confocal microscopy (*n* = 8) [22,23,34,43,45,56,60,62], and protein absorption (*n* = 1) [48].

#### 3.5.8. Mechanical Analysis

The mechanical analyses were performed to evaluate stiffness (*n* = 1) [22], stretch (*n* = 1) [39], strain (*n* = 1) [36], rheology (*n* = 1) [55], compression (*n* = 2) [32,48], volume and sphericity (*n* = 1) [5], gel contraction (*n* = 2) [36,60], and wet weight analysis (*n* = 1) [58].

## 4. Discussion

The use of organoids in periodontal research surpasses 2D cultures and spheroids, primarily due to their ability to better replicate the native tissue complexity, including cellular interactions, extracellular matrix composition, and three-dimensional architecture [63]. This advanced modeling enhances the study of periodontal disease, tissue regeneration, and drug responses, leading to more clinically relevant findings. This review analyzed the challenges and advancements in 3D cell culture organoid models in periodontics, focusing on the cells employed, the biomaterials used for the mimetic extracellular matrix, and techniques for developing 3D cultures. Our findings indicate that most studies used a single cell lineage, with human-derived periodontal ligament cells or human-derived periodontal ligament fibroblasts being the most common. Organoid construction predominantly relied on solid scaffolds, especially synthetic polymers such as poly(caprolactone) (PCL), poly(L-lactide acid) (PLA), and poly(glycolic acid) (PLGA). Natural polymers like collagen and chitosan, along with biocompatible ceramics such as hydroxyapatite and ß-tricalcium phosphate, were also widely employed, often in combination, to enhance structural and functional properties. The most common method for organoid development was direct cell seeding onto scaffold-based extracellular matrix material.

Characterization of periodontal organoids typically assesses cell viability, adhesion, proliferation, migration, differentiation, surface properties, and mechanical behavior. Common assays for viability included MTT [31,34,42,43,44,45], Live/Dead assay [19,20,30,36,51,57], and Alamar Blue [19,41], while cell proliferation was analyzed using Alamar Blue [5,19], MTT [48,49], and CCK-8 [33,51]. Adhesion and migration were evaluated through SEM imaging [31,51] and fluorescence-based analysis [39,49], whereas differentiation was assessed using gene and protein expression markers [5,24,28,32,33,34,37,39,41,45,49,53,56,59]. Gene expression was primarily analyzed using RT-PCR [5,9,24,25,27,28,30,33,34,37,39,43,45,49,53,57,58,59,62] and RNA sequencing [9,32,49]. Protein analysis involved immunohistochemistry [9,21,23,24,35,41,43,47,52,53,54,59,62], ELISA [23,28,44,46,56], immunofluorescence [23,24,25,26,34,49,52], and Western blot [25,50]. Surface characterization was conducted using SEM [31,51], TEM [9,21,56], and confocal microscopy [22,23,34,43,45,56,60,62]. Mechanical properties were assessed using stiffness tests [22], rheological assays [55], and compressive mechanical analysis [32,48], providing valuable insights into the structural, functional, and regenerative potential of periodontal organoids. Nevertheless, the lack of standardized protocols across studies limits comparability and reproducibility. Future work should prioritize direct comparative studies to establish standardizer and reproducible protocols, which are essential for advancing in clinical translation.

Organoids are defined as 3D structures derived from pluripotent stem cells, progenitor cells, and/or differentiated cells, which mimic key structural and functional aspects of their corresponding tissue [58]. Although originally developed from stem cells [63], recent studies have demonstrated organoid formation from differentiated cell lineages [18,64,65,66]. Replicating the periodontal ligament in vitro is challenging due its collagen-rich structure and the difficulty in promoting fiber formation while inhibiting unwanted osteogenic differentiation [49]. The periodontal ligament tissue is heterogenous, comprising fibroblasts, epithelial cells, neural cells, endothelial cells, and undifferentiated mesenchymal cells [64,67]. Although periodontal ligament cells or fibroblasts were most frequently used [5,19,20,21,22,23,24,25,26,27,28,29,30,31,32,33,34,35,36,37,38,39,40,41,42,43,44,45,46,47,54,59,60,61], studies also included periodontal ligament stem cells (*n* = 6) [48,49,50,51,52,53], gingival fibroblasts (*n* = 5) [23,48,54,55,56], immortalized periodontal ligament cells (*n* = 2) [57,58], dental follicle cells (*n* = 2) [58,59], cementoblasts (*n* = 2) [42,59], dental epithelium stem cells (*n* = 1) [9], dental epithelial cells (*n* = 1) [35], dental pulp stem cells (*n* = 1) [53], alveolar bone stem cells (*n* = 1) [53], and alveolar bone cells (*n* = 1) [60]. Only six studies used periodontal ligament stem cells [48,49,50,51,52,53], highlighting an ongoing lack of clarity in stem cell definitions and their applications in culture systems.

Periodontal ligament cells, periodontal ligament fibroblasts, periodontal ligament stem cells, and immortalized periodontal ligament cells may seem similar. To distinguish among these, we categorize them: ref. [1] periodontal ligament cells confine a diverse group of cells within the periodontal ligament, including cementoblasts and osteoblasts, playing key roles in maintaining tissue integrity and homeostasis [65,66]; ref. [2] periodontal ligament fibroblasts are specialized in collagen type I production and extracellular matrix remodeling, and are critical for ligament arrangement [21,34]; ref. [3] periodontal ligament stem cells are a unique multipotent subpopulation with the ability of differentiation (e.g., osteogenic, adipogenic, cementogenic lineage), making them valuable for regenerative therapies [48,49,52]; and ref. [4] immortalized periodontal ligament cells are genetically modified to proliferate indefinitely in vitro, serving as stable models for periodontal research but lacking full native cell functionality [57].

While periodontal ligament cells and fibroblasts were the most used, periodontal ligament stem cells are the most efficient for generating organoids due to their multipotency and regenerative potential. Their ability to differentiate into osteogenic, adipogenic, and cementogenic lineages makes them highly versatile for tissue engineering. Additionally, their self-renewal capacity and interaction with other periodontal cell types enhance their ability to replicate the complex microenvironment of the periodontal ligament in organoid models. Nevertheless, they lack the potential to differentiate into epithelial cell lines with the potential for keratinization.

Most studies focused on human cells for periodontal regeneration, with only four utilizing animal cells [9,35,39,59]. Human cells offer greater physiological relevance and accessibility, particularly from extracted teeth, while animal-derived cells pose challenges in isolation and clinical translatability.

There is currently no standardized protocol for periodontal organoid development, with studies varying in cell types, extracellular matrix composition, and culture methods. Two predominant methods were identified: combining cells with the extracellular matrix components [19,20,23,25,28,31,36,39,62] and seeding cells onto scaffolds [5,9,21,22,24,26,27,29,30,32,33,34,35,37,40,41,42,43,44,45,46,47,48,49,50,51,52,53,54,55,56,57,58,59,61], with only one study [60] combining both. Future research should focus on directly comparing these methods to establish a standardized, reproducible method for periodontal organoid development.

Various biomaterials were used to construct periodontal organoids, including biodegradable polymers such as polycaprolactone (PCL) [22,26,53], poly(hydroxybutyrate-co-hydroxyvalerate) [48], poly(ethylene glycol) (PEG) [5], polyethylene glycol diacrylate (PEGDA) [27], poly(aspartamide) (PASP) [29], poly(L-lactide acid) (PLLA) [32,37], poly(glycolic acid) (PGA) [47], poly(lactic-co-glycolic acid) (PLGA) [59], polyelectrolyte complex (PEC) [61], and polyester [56]. The biomaterial influence load transmission, cell alignment, and collagen fiber organization. Polycaprolactone, in particular, facilitates mechanical load transduction to cells [22], guides cell alignment via 3D-printed tubules [26], and mimics collagen fiber orientation like the periodontal ligament [53].

Several studies using other biodegradable polymers such as poly(hydroxybutyrate-co-hydroxyvalerate), poly(aspartamide) (PASP), PGA, and PEC demonstrated that cells seeded on these scaffolds presented adhesion and proliferation, making them potential candidates for periodontal tissue engineering [29,47,48,56,61]. The use of PEG in one study [27], however, presented divergent results regarding matrix degradability, cell–matrix binding, and rigidity, indicating that further studies are needed to better assess poly(ethylene glycol)’s suitability for 3D culture. PEGDA combined with modified gelatinhyaluronan and hydrogel reduced cell viability [27], potentially due to mechanical stress. In contrast, studies that used PLGA and poly(ester) scaffolds showed the formation of mineral and collagen fibers, important for tissue regeneration [56,59]. Interestingly, PLLA was used in two separate studies [32,37] by the same authors, who demonstrated that the material effectively mimicked the human periodontal ligament response to orthodontic force in vitro. The cells showed osteogenic potential and the appropriate gene expression profile, indicating PLLA’s promise for periodontal regeneration [29,35]. Overall, the use of biodegradable polymers favored the cell adhesion, proliferation, and the formation of mineralized extracellular matrix with collagen fibers, confirming their potential utility in periodontal tissue engineering. Nonetheless, challenges such as scaffold contraction, heterogeneity, limited thickness, rigidity, degradation, and inadequate cell penetration persist. Refinement of biomaterial and the integration of advanced technologies, including 3D bioprinting, are essential to achieve physiologically relevant and functionally integrated periodontal organoids.

Collagen, one of the main components of periodontal ligament fibers, was widely used due to its biocompatibility and ability to support cell adhesion, migration, proliferation, and differentiation [20,21,25,34,35,36,39,41,46,47,50,54,57,60]. Collagen supported cell proliferation regardless of the cell line used [34,36,44,60] and enhanced the formation of a mineralized matrix [34]. Several genes were upregulated in studies using collagen, including COL1 [5,24,28,32,34,37,39,41,45,49,53,56,59], ALP [5,20,22,29,30,31,33,34,37,42,43,45,46,50,51,56], cytokeratin [35], CK8+18+19 [35], ILK [25,41], periostin [41], Runx-2 [39,41], PDGF [44], and IL-6 [46], indicating the collagen positive impact on key regenerative processes in periodontal tissues. These findings suggest that collagen is particularly beneficial for periodontal applications due to its structural similarity to natural ligament components, making it an ideal material for mimicking the native extracellular environment.

Additional biomaterials, including gelatin [19,27,28,49,51], chitosan [30,42,43,44,45], hydroxyapatite [24,33,40,45,51,52,53], alginate [19,25,51], calcium phosphate [33,43,52], fibrin [23], and Matrigel^®^ [9] were also employed, often in combination with other materials. Chitosan was particularly effective in supporting periodontal ligament cell proliferation within the scaffolds, making it an excellent candidate for regenerative applications [43,45]. Hydroxyapatite and calcium phosphate were particularly distinguished for their ability to promote mineralization, key for bone regeneration in the periodontal context.

Among the materials studied for ligament periodontal organoid formation, collagen and biodegradable polymers stand out as the most promising. Collagen closely mimics the native extracellular matrix, supporting cell adhesion, migration, proliferation, and differentiation, while enhancing mineralized matrix formation [34,36,41,50]. Among biodegradable polymers, PCL and PLLA exhibited excellent mechanical properties, the ability to align collagen fibers, and the capacity to replicate periodontal ligament responses to mechanical forces [22,26,32,37,53]. These materials provide a structurally and biologically favorable environment, making them ideal for periodontal tissue engineering and organoid development.

Despite the significant research on organoids for tissue regeneration, few studies have used organoids to analyze the interaction between microorganisms and periodontal cells. Two studies in this review examined these interactions [23,62]. Golda et al. [62] created an organoid model using gingival fibroblasts and telomerase-immortalized gingival keratinocytes stratified on a Matrigel^®^ matrix. This model was infected with Porphyromonas gingivalis and herpes simplex virus 1 (HSV-1), and it was observed that the organoid resembles real gingival tissue, demonstrating its potential for studying host–pathogen interactions. This model holds promise for exploring therapies for periodontal diseases. Another study by Makkar et al. [23] used gingival fibroblasts and periodontal ligament fibroblasts mixed with a fibrin–based hydrogel matrix to investigate interactions with *Streptococcus mitis* and *Streptococcus oralis* biofilm. The authors concluded that organoids provide an excellent platform for such studies, although longer-term analyses are required to understand the full potential of these 3D cultures. Despite the promise of these initial studies, more research is needed to explore how organoids can be used not only for periodontal regeneration but also for preventing microbial destruction of periodontal tissues. Studying periodontal microbiology in an organoid model provides a physiologically relevant platform that closely mimics native periodontal tissues. Unlike traditional in vitro models, organoids enable dynamic interactions between host cells and microbial communities, offering deeper insights into host–microbe interactions. This controlled and reproducible system allows for the study of infection mechanisms, inflammatory responses, and the evaluation of potential therapeutic strategies for periodontal diseases.

The construction of ligament periodontal tissue organoids presents challenges due to the complex cellular architecture and cell–matrix interactions. Most (*n* = 24) of the 44 articles included in this review reported limitations [5,19,23,26,27,28,29,30,33,34,35,36,39,41,44,50,51,54,55,56,57,59,60,61], including material contraction [23,56,60], heterogeneity due differences in material composition, scaffold architecture, mechanical stimulation, and cell quantification methods [5,19,26,36,55,61], thickness [36,57], stiffness [27,29], degradation [19], and cell penetration [19,51]. After cell incorporation, the significant difficulties encountered were related to cellular proliferation [19,28,33,35,41,54,61], orientation [56], morphology [61], and detachment [30,39,56]. Divergences in ALP activity [33,34,56], mineralized tissue formation [50,59], and gene expression assays [44] highlight the need for methodological standardization [30,31,52]. Additionally, differences were observed in the induction of mineralized tissue formation, suggesting that certain cell types or experimental conditions influenced this process differently [50,59]. Lastly, gene expression analysis assays revealed inconsistencies in the expression of key markers, reflecting potential variations in cellular profiles and regenerative mechanisms [44]. These divergences may stem from methodological differences. Another difficulty related was the selection of appropriate positive control genes for use in 3D culture [27]. To overcome these limitations in periodontal organoid development, optimizing the extracellular matrix composition and scaffold properties is crucial. Using collagen-based hydrogels or hybrid biomaterials, such as PCL combined with gelatin or fibrin, could improve cell adhesion, proliferation, and orientation while reducing contraction and detachment issues. Additionally, bioreactors and dynamic culture systems may enhance nutrient diffusion, matrix penetration, and cellular organization, addressing heterogeneity and thickness concerns.

Although 3D cell culture systems have advanced significantly, they remain more complex and costly than traditional 2D cultures, especially when multiple cell lines are involved. This underscores the need for improved methodologies to enhance accessibility and efficiency. Despite these challenges, periodontal organoids hold immense potential to revolutionize tissue regeneration, personalized therapies, drug testing, and disease modeling by accurately mimicking the native periodontal environment. Furthermore, bioprinting has emerged as a promising tool to enhance organoid fabrication, allowing precise control over cell distribution, extracellular matrix composition, and scaffold architecture, ultimately improving tissue organization and functional integration [68,69]. To fully exploit this potential, future research should focus on optimizing organoid preparation methods, refining biomaterials, integrating vascularization and immune components, and expanding their application to study pathogen interactions. Moreover, standardizing protocols and fostering collaboration across research groups will be key to accelerating progress and ensuring the successful clinical translation of periodontal organoid technology.

## 5. Conclusions

In conclusion, this review highlights the predominant use of periodontal ligament cells and fibroblasts, with periodontal ligament stem cells being the most effective for organoid formation due to their multipotency, despite their limited differentiation potential into epithelial cells poses a challenge for fully replicating the heterogeneous periodontal tissue. Scaffolds, primarily biodegradable polymers like polycaprolactone and poly(L-lactide acid), and natural biomaterials such as collagen and chitosan, were essential for mimicking the extracellular matrix and supporting cellular functions. However, inconsistencies in methodologies, scaffold properties, and cellular responses underscore the need for standardized protocols. Future research should focus on optimizing biomaterials, integrating vascularization and immune components, and leveraging bioprinting technology to enhance organoid development. Despite current challenges, periodontal organoids hold significant potential for advancing tissue engineering, disease modeling, and personalized regenerative therapies.

## Figures and Tables

**Table 1:** Summary of descriptive characteristics of included studies on the solid scaffolds category (*n* = 21). Legend: 3D—three-dimensional; ABBS—avidin biotin binding system; ALP—alkaline phosphatase; BCP—biphasic calcium phosphate; bFGF—basic fibroblast growth factor; BSP—bone sialoprotein; BTCP—beta-tricalcium phosphate; CAP—cementum attachment protein; CEMP1—cementum protein 1; COL1—collagen type I; DAPI—(4′,6-diamidino-2-phenylindole); d-ECM—decellularized extracellular matrix; DNA—deoxyribonucleic acid; ECM—extracellular matrix; ELISA—enzyme-linked immunosorbent assay; FGF-2—fibroblast growth factor 2; GAM—gene-activated matrix; HA/Chitosan scaffold—chitosan and hydroxyapatite scaffold; H&E—hematoxylin and eosin; HGFs—human gingival fibroblasts; hPdLLT—human periodontal ligament-like tissue; hPDLC—human periodontal ligament cells; hPLF—human periodontal ligament fibroblast; IL-6—Interleukin 6; MTT—(3-(4,5-dimethylthiazol-2-yl)-2,5-diphenyltetrazolium bromide); NANOG—homeobox protein NANOG; n-HA scaffold—nanohydroxyapatite scaffold; OCCM/PLGA—cementoblast cell line with poly(lactic-co-glycolic acid); OCT4—octamer-binding transcription factor 4; OPG—osteoprotegerin; OPN—osteopontin; P(HB-50HV)—poly(hydroxybutyrate-co-hydroxyvalerate) with 50% HV content; PCL/HA scaffold—poly(caprolactone) and hydroxyapatite; PDGF—platelet-derived growth factor; PDL—periodontal ligament cells; PDLSC—periodontal ligament stem cells; PEC—polyelectrolyte complex; PLAP-1—placental alkaline phosphatase 1; PLGA—poly(lactic-co-glycolic acid); PLLA—poly(L-lactide acid); PLSCs—periodontal ligament stem cells; RANKL/BMP-2—receptor activator of nuclear factor kappa-B ligand/bone morphogenetic protein 2; RT-PCR—reverse transcription polymerase chain reaction; RUNX2—Runt-related transcription factor 2; SEM—scanning electron microscopy; SOX2—SRY-box transcription factor 2; STRO-1—stromal precursor antigen-1; TEM—transmission electron microscopy.

Author (Year)	Cell Lineage (Origin)	Extracellular Matrix Composition	Organoid Preparation	Characterization Methods	Results	Main Findings
Gauthier, et al. (2023) [22]	Periodontal ligament cells (human)	Fibrous polycaprolactone scaffold	Cells were seeded into the scaffolds after their synthetize through electrospinning.	- Mechanical characterization - Confocal microscopy - ALP - Inflammatory biomarkers	- In static culture, surface cell networks were observed with increased ALP and IL6 expression. - In dynamic culture, fewer surface cells were present with decreased ALP and IL6 expression. - Stiffness decreased in both conditions.	Polycaprolactone was a suitable material to mimic the periodontal ligament collagen bundles.
Phuegyod, et al. (2023) [48]	Gingival fibroblasts + Periodontal ligament stem cells (human)	Poly(hydroxybutyrate-co-hydroxyvalerate) scaffold	The scaffolds were prepared as a cylindrical shape, and cells were seeded later.	- Compressive mechanical analysis - Protein absorption - Cell proliferation	- P(HB50HV) scaffolds were different in compressive modulus and had the lowest stiffness among all scaffolds. - HGF and PDLSC adhered to the scaffold and exhibited the highest proliferation.	Microbial-derived P(HB-50HV) scaffold can be used as a biomaterial for periodontal tissue engineering and stem cell applications.
Ono, et al. (2021) [24]	Periodontal ligament cells (human)	Tubular scaffold with titanium and hydroxyapatite core	Single-cell spheroids were picked and inserted into the 3D positions of the needle array to form a pre-designed 3D tubular model.	- RTPCR - H&E - Immunohistochemical staining - Masson’s staining - Picrosirius red - Immunofluorescence - Cell distribution	- Angiogenesis, cementum, and - bonerelated genes increased in the three complexes compared with - monolayercultured cells. - Abundant collagen fibers for the above markers were confirmed in the three complexes.	Exhibited high cell viability, included abundant collagen fibers, and expressed factors strongly associated with PDL tissue.
Lin, et al. (2021) [57]	Immortalized periodontal ligament cells (human)	3D-printed collagen-based scaffold	Cells were seeded onto the collagen microfibrous scaffolds.	- Live/Dead assay - DAPI cell counting - RTPCR	- Collagenbased microfibers were successfully fabricated. - Cytoskeleton expansion, adhesion, and viability were greater. - Cyclin D, Ecadherin, and periostin were upregulated on the waveform microfibers.	Preserved cell viability and promoted healing and regeneration under shear stress.
Kim, et al. (2020) [26]	Periodontal ligament cells (human)	3D-printed poly-ε-caprolactone scaffold	Poly-ε-caprolactone solution was cast into the support material mold. Cells were seeded into all scaffolds.	- Surface characterizations - Immunofluorescence	- Four microgroove intervals fabricated. - Highest alignment at 25 µm grooves; alignment decreased with smaller intervals. - Greater elongation and lower circularity at 25 and 19 µm compared to 12 and 6 µm. - Lowresolution grooves guided cells better than smooth highresolution surfaces.	Controllable microgroove intervals can specifically organize human PDL cells by 3D printing. Surface topography can angularly guide human PDL cells.
Shi, et al. (2018) [52]	Periodontal ligament stem cells (human)	Porous β-tricalcium phosphate and hydroxyapatite scaffold	Cell suspension was dropped onto the BCP by means of the pipetting technique.	- Immunohistochemical staining - Osteogenic differentiation - Cell seeding - Immunofluorescence - MicroCT - Masson staining	- PDLSC were positive for STRO1 and vimention, while negative for cytokeratin. - PDLSC had the capacity to undergo osteogenic differentiation in vitro. - When seeded on the BCP, PDLSC exhibited great viability.	The incorporation of PDLSC and the improved BCP significantly restored the lost periodontium. BCP is one of promising bioactive materials for periodontal tissue regeneration.
Yan, et al. (2018) [30]	Periodontal ligament cells (human)	Chitosan film scaffold	Chitosan scaffold was prepared, and the cells were seeded.	- Live/Dead - RTPCR - Colonyforming efficiency - ALP - Calcium content	- Decreased proliferation upon spheroid formation. - Expression of genes was significantly higher comparison with cells cultured in a monolayer.	Compared to cells cultured in monolayer, periodontal ligament cells did not proliferate, but exhibited higher self-renewal gene expression, colony-forming unit, and osteogenic capacity.
Liao, et al.(2016) [32]	Periodontal ligament fibroblasts (human)	Porous poly(L-lactide) scaffold	Cells were seeded and centrifuged into a round-shaped PLLA matrix.	- Compressive mechanical analysis - SEM - Caspase3/7 activity - RTPCR	- High viability up to 14 days. - RANKL/BMP2 load and timedependent peaks. - ALP/PLAP1 increased at low load (5 g/cm^2^). - FGF2 and OPG show a timedependent increase. - COL1 progressive rise over time.	The in vitro hPdLLT model system effectively mimicked the reaction and gene expression of the human periodontal ligament in response to orthodontic force.
An, et al. (2015) [33]	Periodontal ligament cells (human)	β-tricalcium phosphate and hydroxyapatite scaffold	Cells were seeded on the scaffolds.	- Alizarin red - Cell proliferation - SEM - ALP - RTPCR	- Good proliferation and survival on collagen scaffold. - Fibroblastlike alignment within scaffold. - Increased COL1, ALP, and CEMP1. - Positive Alizarin red staining.	Three-dimensional porous BCP scaffolds can stimulate the osteoblastic differentiation of hPDLC in the presence and absence of osteogenic inducer.
Lee, et al. (2014) [53]	Dental pulp stem cells + Periodontal ligament stem cells + Alveolar bone stem cells (human)	3D-printed poly(caprolactone)-hydroxyapatite scaffold	PCL/HA scaffolds were fabricated, and the cells were seeded.	- RTPCR - Histomorphometric analysis - Immunohistochemical staining	- Cells maintained fibroblastlike shape and spread well. They demonstrated high survival and proliferation within dECM. - Upregulation of COL1, ALP, and periostin. - Positive calcium deposition.	Method for the regeneration of multiphase periodontal tissues by spatiotemporal delivery of multiple proteins.
Liao, et al. (2013) [37]	Periodontal ligament fibroblasts (human)	Porous poly(L-lactide) scaffold	PLLA matrix was prepared, and the cells were seeded.	- SEM - Cell proliferation - Picrosirius red - RTPCR - ALP	- High survival and compact spheroid formation. - Expression of NANOG, OCT4, and SOX2. - Upregulation of osteogenic and cementogenic markers. - Positive calcium deposition.	The results indicated in vitro human periodontal ligament-like tissue, and it could be used in future periodontal ligament tissue engineering.
Jang, et al. (2011) [40]	Periodontal ligament fibroblasts (human)	Nanohydroxyapatite scaffold with avidin biotin binding system	Cells were loaded onto the scaffolds.	- SEM	- ABBS method showed significantly higher fibroblast attachment to nHA scaffolds than static or agitation methods. - SEM showed no significant differences among groups. - Adhesion strength improved with ABBS.	The high-affinity ABBS enhances the ability of periodontal ligament fibroblasts to attach to three-dimensionally constructed n-HA scaffolds.
Berahim, et al. (2011) [41]	Periodontal ligament fibroblasts (human)	Collagen and poly(glycolic acid)-based membrane	Membrane squares were placed, and periodontal spheroids were pipetted into each ring.	- Alamar Blue - Immunohistochemical staining - SEM	- Adhesion and proliferation were higher on collagen than PGA. - The activity of cells increased on both, but collagen was superior until day 15. - The protein expression of collagen I, periostin, and Runx2 on both, but it was stronger on collagen.	Were able to grow three-dimensionally on the biologic membranes and may have the potential to be used together with guided tissue regeneration approaches.
Akman, et al. (2010) [42]	Periodontal ligament cells + Cementoblasts (human)	Chitosan and hydroxyapatite scaffold loaded with basic fibroblast growth factor	Porous chitosan scaffolds were prepared, and the cells were added drop-by-drop with a micropipette.	- MTT - Von Kossa - ALP	- Growth factor release was sustained up to 7 days and lasted longer in HAchitosan. - Cell proliferation was enhanced in both PDL cells and cementoblasts, with the highest levels observed in HAchitosan with bFGF. - Mineralization and ALP activity were stronger in bFGFloaded HAchitosan than in chitosan.	bFGF-loaded HA-chitosan scaffolds supported the cellular structure, proliferation, and mineralization.
Liao, et al. (2010) [43]	Periodontal ligament cells (human)	β-tricalcium phosphate and chitosan scaffold	Cell suspensions were seeded into each scaffold.	- SEM - Confocal microscopy - MTT - RTPCR - H&E - Immunohistochemical staining - Modified Gomori’s ALP	- βTCP/chitosan scaffold showed higher cell adhesion, proliferation, and migration than pure chitosan. - Upregulated BSP and CAP expression in vitro and increased ALP and OPN expression in vivo. - Vascular ingrowth and ECM formation indicated enhanced differentiation.	The scaffold could promote the differentiation of hPDLC towards osteoblast and cementoblast phenotypes.
Peng, et al. (2009) [44]	Periodontal ligament cells (human)	Chitosan and collagen scaffold	Chitosan/collagen composite scaffold was prepared, and the cell suspension was added.	- SEM - Hoechst 33258 - MTT - Luciferase reporter gene assay - Histomorphometrical analysis	- Chitosan/collagen GAM with PDGF nanoparticles sustained DNA release for over six weeks. - Enhanced PDL cell adhesion, proliferation, and tissuelike structure formation. - PDGF expression and cell density were significantly higher than with naked plasmid scaffolds.	-PDLC achieved high proliferation and maintained a fibroblast figure, and the periodontal connective tissue-like structure formed in the scaffolds. The novel gene-activated matrix (GAM) had potential in the application of periodontal tissue engineering.
Zhang, et al. (2007) [45]	Periodontal ligament cells (human)	Porous nanohydroxyapatite and chitosan scaffold	HA/chitosan composites were mixed, and cells were suspended and poured onto each scaffold.	- MTT - SEM - ALP - RTPCR - Confocal microscopy - H&E	- 1% HA/chitosan scaffold showed better cytocompatibility than pure chitosan. - Enhanced PDL cell adhesion, proliferation, and expression of type I collagen and ALP. - Scaffold supported vascular ingrowth, ECM formation, and slower degradation in vivo.	1% HA/chitosan scaffold had potential applications as biomaterials in periodontal tissue engineering.
Wang, et al. (2005) [47]	Periodontal ligament cells (human)	Poly(glycolic acid) scaffold	PGA scaffolds were prepared and PDL cells were seeded.	- SEM - Immunohistochemical staining	- PGA scaffolds supported strong adhesion and proliferation. - PDL cells secreted abundant extracellular matrix and expressed type I collagen.	May serve as a viable approach for promoting periodontal tissue regeneration and provides a possibility of PDL regeneration on dental implants.
Jin, et al. (2003) [59]	Cementoblasts + Periodontal ligament fibroblasts + Dental follicle cells (mice)	Poly(lactic-*co*-glycolic acid) scaffold	Scaffolds were prepared, and cells were added.	- RTPCR - Immunohistochemical staining - Histomorphometric analysis	- PLGA scaffolds supported adhesion of cementoblasts, PDL cells, and follicle cells. - Only cementoblastseeded scaffolds promoted mineral formation. - OCCM/PLGA scaffolds expressed collagen I, collagen XII, BSP, and osteocalcin and showed progressive mineralization in vivo.	Delivery of selected cells via PLGA scaffolds may serve as a viable approach for promoting periodontal tissue regeneration.
Hillmann, et al. (1999) [56]	Gingival fibroblasts (human)	Polyester with polypropylene scaffold coated with fibronectin	Polyester with polypropylene scaffold was pretreated and coated with fibronectin. Cells were seeded onto the polyester mesh.	- H&E - ELISA - ALP - Immunoelectron microscopy - Scanning electron microscopy (SEM) - Confocal microscopy - TEM - Hemalum staining	- Scaffold showed uniform porosity, good biocompatibility, and supported PDL cell adhesion and proliferation. - Cells expressed type I collagen and fibronectin. - Scaffold promoted new bone, cementum, and PDL formation in vivo.	A dynamic model for performing studies on the interactions of cultured cells with extracellular matrix molecules, the pathogenesis of inflammatory processes, and the interactions with biomaterials.
Hamano, et al. (1998) [61]	Periodontal ligament fibroblast (human)	Polyelectrolyte complex	Both the polyanions and the synthetic polycations were dissolved and mixed. Cells were seeded later.	- SEM - Alizarin red - Phase contrast microscopy	- Polyelectrolyte complexes (PECs) modulated PDL fibroblast morphology, proliferation, and differentiation. - PECs with carboxymethyl groups induced cell aggregation, 3D structures, and mineralization. - PECs with sulfate groups promoted spreading and proliferation without mineralization.	PECs affected the cell cycle and promoted proliferation and differentiation of hPLF.

**Table 2:** Summary of descriptive characteristics of included studies on the hydrogel category (*n* = 15). Legend: AB/JE—ameloblasts markers/enamel-bound junctional epithelium; Alpha-SMA—alpha-smooth muscle; ALP—alkaline phosphatase; BSP—bone sialoprotein; CaSO4—calcium sulfate; CAP—cementum attachment protein; CEMP1—cementum protein 1; COL1A1—collagen type I alpha 1 chain; COL3A1—collagen type III alpha 1 chain; CREB3L3—cyclic AMP-responsive element-binding protein 3-like 3; CTGF—connective tissue growth factor; DAPI—(4′,6-diamidino-2-phenylindole); d-Oxd or the d-pGlu [Oxd = (4R,5S)-4-methyl-5-carboxyl-oxazolidin-2-one, pGlu = pyroglutamic acid]; DE/DESC—dental epithelium and dental epithelium stem cells; DMEM—Dulbecco’s Modified Eagle Medium; DNA—deoxyribonucleic acid; ECM—extracellular matrix; EdU incorporation—5-ethynyl-2′-deoxyuridine; ELISA—enzyme-linked immunosorbent assay; EMD—enamel matrix derivative; ERN2—endoplasmic reticulum to nucleus signaling 2; FGF-2—fibroblast growth factor 2; Gelin-S—thiol-modified gelatin; GelMA—gelatin methacryloyl; hPDLC—human periodontal ligament cells; hPDLF—human periodontal ligament fibroblasts; hPLSC—human periodontal ligament stem cells; ILK—integrin-linked kinase; LDH—lactate dehydrogenase; MMP—matrix metalloproteinase; MTT—(3-(4,5-dimethylthiazol-2-yl)-2,5-diphenyltetrazolium bromide); NAD(P)H—nicotinamide adenine dinucleotide phosphate; NaOH—sodium hydroxide; OCN—osteocalcin; OPG—osteoprotegerin; PDL—periodontal ligament; PDLC—periodontal ligament cell; PDLC—periodontal ligament cells; PDLSC—periodontal ligament stem cells; PEGDA—polyethylene glycol diacrylate; PI3K—phosphatidylinositol 3 kinase; RhPAI-1—Recombinant Human Plasminogen Activator Inhibitor-1; RNA-seq—RNA sequencing; RT-PCR—reverse transcription polymerase chain reaction; RUNX2—Runt-related transcription factor 2; SEM—scanning electron microscopy; shRNA—short hairpin ribonucleic acid; SP7—Sp7 transcription factor (osterix); TEM—transmission electron microscopy; TGFB-1—transforming growth factor beta 1; TIMP-1—tissue inhibitor of metalloproteinases 1; Twist1—Twist family bHLH transcription factor 1; UPR—unfolded protein response; UV light—ultraviolet light; YAP-1/TWIST1—Yes1-associated transcriptional regulator/Twist1 transcription factor; Yap1—Yes1-associated transcriptional regulator.

Author (Year)	Cell Lineage (Origin)	Extracellular Matrix Composition	Organoid Preparation	Characterization Methods	Results	Main Findings
Schweinitzer, et al. (2024) [19]	Periodontal ligament fibroblasts (human)	Gelatin methacryloyl (GelMA ^ ® ^ ) or sodium alginate hydrogels	Cells were mixed into the hydrogels.	- Live/Dead - Alamar Blue - Cell proliferation - DAPI cell counting	- Fibroblasts spread and proliferated in GelMA but remained rounded with reduced growth in alginate. - Alamar Blue overestimated proliferation compared to PicoGreen. - Image analysis confirmed viability but was less accurate.	Discrepancies between these assays, attributable to their mechanisms of action and their different protocols.
Chang. (2023) [20]	Periodontal ligament fibroblasts (human)	Collagen hydrogels	Cells were mixed into the hydrogels.	- Strain test - Live/Dead assay - AlphaSMA - ALP - Histomorphometric analysis	- Good strength and supported cell differentiation into myofibroblasts and preosteoblasts. - Reduced epithelial downgrowth, residual bone defects, and promoted welloriented PDL and alveolar bone regeneration in vivo.	Periodontal tissue regeneration was achieved. Hydrogels incorporating cells were superior compared to hydrogels.
Hermans, et al. (2023) [9]	Dental epithelium stem cells (mice)	Hydrogels (Matrigel^®^)	Cells seeded into the hydrogel droplets	- Immunohistochemical staining - EdU incorporation - TEM - RTPCR - RNAseq - Organoid survival - Differentiation potential	- Organoids showed longterm expandability and expression of dental stem cell markers. - Differentiated into ameloblastlike cells and, when combined with pulp stem cells, also into odontoblastand cementoblastlike cells. - Capacity to survive and maintain differentiation in vivo.	A valuable tool to study mouse tooth DE/DESC, dental epithelial–mesenchymal interactions, and AB/JE differentiation while allowing further elucidation of tooth-type-specific features.
Liang, et al. (2022) [49]	Periodontal ligament stem cells (human)	Nanofibrous gelatin	Scaffold synthetized through electrospinning, and cells were seeded onto gelatin scaffolds.	- Cell proliferation - Cell adhesion - Cell migration - Immunofluorescence - Picrosirius red - Alizarin red - Yap1 and Twist1 inhibition - RTPCR - RNAseq	- PDLSC migration and alignment, leading to wellorganized PDLlike fiber bundles with expression of collagen I, collagen III, and periostin. - Inhibited osteogenic differentiation by downregulating OCN, Runx2, and Sp7 through the Yap1/Twist1 pathway.	Tubular matrix mimicked the physical architecture and chemical compositions of the ECM of PDL, controlled and aligned PDLSC migration inside the tubules, and inhibited the osteogenesis.
Yasunaga, et al. (2022) [50]	Periodontal ligament stem cells (human)	Collagen hydrogel	The prepared collagen gel mixtures were applied, and the spheroids transferred to the mixtures in the plates.	- Western blot - Immunohistochemical staining - ALP - Alizarin red	- Showed enhanced cementogenic differentiation with strong upregulation of CEMP1 and CAP. - Interacted with collagen ECM, disaggregated, and promoted cell migration, leading to higher cementoblastic differentiation than 2D or untreated 3D cultures.	RhPAI-1 treatment of embedded hPLSC spheroids enhanced the cementogenic.
Tian, et al. (2021) [51]	Periodontal ligament stem cells (human)	Sodium alginate, gelatin, and nano-hydroxyapatite mixed into a hydrogel	The hydrogels were prepared, and cell was added.	- Live/Dead assay - Cell adhesion - Cell proliferation - ALP	- Improved mechanical strength, lower swelling, and good porosity compared to SA alone. - PDLSC demonstrated high viability, stronger adhesion, and enhanced proliferation. - ALP activity and osteogenic differentiation were significantly higher in the composite scaffold.	Hydrogels showed effective stimulation for cell survival, proliferation, and osteoblast differentiation.
Fraser, et al. (2021) [5]	Periodontal ligament cells (human)	Poly(ethylene glycol) hydrogel	One-milliliter syringe was used as a hydrogel mold. Cells were pipetted into cylindrical syringe molds and formed under UV light for 3 min.	- Cell viability - Cell proliferation - RTPCR - ALP - Immunocytochemical staining - Volume and sphericity - ECM mineralization	- Showed interconnected porous structures and good biocompatibility. - Enhanced cell adhesion, spreading, and proliferation, and significantly upregulated osteogenic markers ALP, Runx2, and OCN. - Promoted new bone formation in vivo.	Hydrogels expressed key PDL matrix genes but did not the mineralization.
Zou, et al. (2021) [25]	Periodontal ligament cells (human)	Collagen–alginate hydrogel	Type I collagen solution, DMEM, sodium alginate solution, CaSO_4,_ and NaOH solution were mixed. The hPDLC suspension was added. The mixed solution was added to the pre-designed mold.	- DAPI - Transduction of ILK short hairpin (shRNA) lentiviral vectors - PI3K - Immunofluorescence - RTPCR - Western blot	- High viability, spreading, and proliferation. - ALP activity increased. - Mineralization occurred mainly in stiffer degradable hydrogels with inductive media.	The static compressive stress can induce autophagy in hPDLC regulated by ILK and PI3K and upregulate ILK expression in a PI3K-dependent manner.
Firth, et al. (2020) [27]	Periodontal ligament cells (human)	Thiol-modified hyaluronan–gelatin, poly(ethylene glycol) diacrylate cross-linked hydrogel	The cells were seeded on thiol-modified hyaluronan–gelatin, polyethylene glycol diacrylate (PEGDA) cross-linked hydrogel.	- Cell viability - Caspase 3/7 activity - RTPCR	- Reduced viability without increasing caspase3/7 activity. - CREB3L3 and ERN2 were upregulated.	Three-dimensional mechanical strain PDL cell culture models offer a method to study the role of endoplasmic reticulum stress and unfolded protein response (UPR).
Saminathan, et al. (2013) [28]	Periodontal ligament cells (human)	Hyaluronan–gelatin hydrogel film	Glycosil and Gelin-S were mixed and PDL cells added.	- Cell viability - Cell proliferation - RTPCR - ELISA	- PDL cells organized into bilayers and maintained high viability. - CTGF and FGF2 enhanced proliferation. - Multiple genes, including RUNX2, COL1A1, COL3A1, MMPs, TIMP1, TGFB1, and OPG, were upregulated, while RANKL remained stable.	Cells organized into a bilayer and stimulated cell proliferation. Suitable for investigating the pathogenesis of periodontal diseases.
Hegedűs, et al. (2019) [29]	Periodontal ligament cells (human)	Poly(aspartamide)-based hydrogel	The poly(aspartamide) gel discs were prepared, and the cells were seeded.	- Cell viability - Phase contrast microscopic - ALP	- PDLC attached, proliferated with higher viability. - Free thiol groups promoted adhesion, longterm proliferation, and migration into the matrix. - High thiol density enhanced spontaneous and induced osteogenic differentiation, with increased ALP activity.	PDLC attaches and grows on hydrogels. -The presence of thiol groups enhances viability and facilitates the osteogenic direction of the differentiating cells.
Zanna, et al. (2017) [55]	Gingival fibroblasts (human)	Pseudopeptides gelators containing the d-Oxd or the d-pGlu [Oxd = (4R,5S)-4-methyl-5-carboxyl-oxazolidin-2-one, pGlu = pyroglutamic acid] moiety and selected amino acids hydrogel	The mixture was stirred until gel formation, and the cells were seeded.	- Rheological analyses - Lactate dehydrogenase (LDH) assay - NAD(P)Hdependent cellular oxidoreductase mitochondrial enzyme activity	- Strong mechanical properties, injectability, and rapid recovery after shear stress. - High viability and proliferation over 7 days, with gelator toxicity eliminated upon selfassembly.	Hydrogels allow the growth of encapsulated cells with very good viability. These hydrogels may be used for 3D cell culture in regenerative medicine.
Mino, et al. (2017) [31]	Periodontal ligament cells (human)	Mebiol Gel (hydrogel)	hPDLC cells were mixed with Mebiol Gel.	- ALP - Surface characterization - SEM - Cell adhesion - MTT	- Cells adhered to sterilized root surfaces in 3D culture, forming projections and starshaped morphology. - ALP activity increased in calcification medium, and mineralized nodules appeared from day 4. - MTT assay confirmed a tenfold increase in cell activity by day 21	PDL cells can adhere to sterile root surfaces and can serve as scaffolds for PDL regeneration
Heckler, et al. (2013) [36]	Periodontal ligament fibroblasts (human)	Type I bovine collagen hydrogel	hPDLF cells were mixed with sterile PureCol (type I bovine collagen ) and polymerized.	- Finite element analysis - Live/Dead assay - PicoGreen - Gel contraction - Picrosirius red	- High viability, proliferation, and contractility up to 14 days. - Collagen fibers became reorganized and aligned, mimicking in vivo PDL. - Application of tensile forces maintained cell viability and increased DNA content, while compressive forces led to decreased proliferation.	3D model of orthodontic tooth movement offers promise for use as a model system in future studies.
Oortgiesen, et al. (2012) [39]	Periodontal ligament fibroblasts (rats)	Type I rat collagen hydrogel	Cells were mixed with collagen hydrogel.	- Mechanical stimulation - Cell distribution - Cell orientation - Cell number analysis - RTPCR	- Cells remained randomly oriented, while mechanical loading increased cell numbers and induced perpendicular alignment to strain. - EMD enhanced proliferation and upregulated BSP and collagen I but downregulated Runx2.	3D model to mimic an authentic PDL space, and it also provided a useful and well-controlled approach to study cell response to mechanical loading and other stimuli.

**Table 3:** Summary of descriptive characteristics of included studies on protein-based category (*n* = 8). Legend: 2D—two-dimensional; 3D—three-dimensional; ALP—alkaline phosphatase; cDNA—complementary DNA; CK19—cytokeratin 19; COL1—collagen type I; CTEs—periodontal connective tissue equivalents; DMEM—Dulbecco’s Modified Eagle Medium; ELISA—enzyme-linked immunosorbent assay; FBS—fetal bovine serum; FDC-SP—follicular dendritic cell-secreted protein; GE—gingival epithelium; GF—gingival fibroblasts; hPDLF—human periodontal ligament fibroblast; hTERT—human telomerase reverse transcriptase; HSV-1—herpes simplex virus type 1; IL-6—Interleukin 6; JE—junctional epithelium; Ki-67 antigen detection—cell proliferation-associated antigen of antibody Ki-67; ODAM—odontogenic ameloblast-associated protein; OPG—osteoprotegerin; OPN—osteopontin; OTG—organotypic 3D gingival model; PEG—polyethylene glycol; RANKL—receptor activator of nuclear factor kappa-B ligand; RT-PCR—reverse transcription polymerase chain reaction; RUNX-2—Runt-related transcription factor 2; SE—sulcular epithelium; SEM—scanning electron microscopy.

Author (Year)	Cell Lineage (Origin)	Extracellular Matrix Composition	Organoid Preparation	Characterization Methods	Results	Main Findings
Golda, et al. (2024) [62]	Telomerase-immortalized gingival keratinocytes (human) + Immortalized gingival fibroblasts-hTERT (human)	Hydrogel	Gingival fibroblasts were mixed with hydrogel. Then, keratinocytes were added to the top.	- Immunohistochemical staining - Confocal microscopy - RTPCR - Titration techniques - Colonyforming	- *P. gingivalis* caused deeper invasion and biofilm formation depending on strain. - HSV1 showed efficient replication with a peak at 72 h. - Metronidazole reduced bacteria and acyclovir reduced virus, most effective when applied early. - *P. gingivalis* increased IL6 and IL8, while HSV1 increased interferons.	The OTG model resembled the morphology of the human gingiva; the pathogens penetrated deep into the tissue, forming a biofilm on the cell surface.
Mahdi Souzani, et al. (2023) [21]	P eriodontal ligament fibroblasts (human)	Bovine type I collagen	Cells were mixed into bovine type I collagen	- Immunohistochemical staining - TEM - SEM - Protein expression	- Homogeneous hPDLF distribution, spindlelike morphology, and higher Factin and periostin expression. - Thicker bundled fibers and stronger cell–matrix interaction. - High pressure reduced cell distribution, induced multinucleated cells, and increased RANKL/OPG expression.	This is the first to investigate the effect of hydrostatic pressure on a 3D model of hPDLF, showing that low pressure promotes even distribution of cells and greater expression of F-actin and periostin, while high or no pressure reduces these effects.
Makkar, et al. (2022) [23]	Gingival fibroblasts + Periodontal ligament fibroblasts (human)	Human fibrin-based matrix	PEG–fibrinogen solution was mixed with fibroblast cell suspension.	- Confocal microscopy - Immunohistochemical staining - Immunofluorescence - Hybridization - ELISA	- *Streptococcus mitis* and *S. oralis* in planktonic state triggered little to no immune response, while their biofilms induced moderate IL6 and IL8 secretion. - *Fusobacterium nucleatum* caused strong cytokine production. - Gingival constructs secreted more IL6, whereas periodontal constructs secreted more IL8.	The gingival and periodontal CTEs exhibited differential responses to various bacterial challenges.
Alves, et al. (2015) [34]	Periodontal ligament fibroblast (human)	Collagen solution	Scaffolds were prepared by mixing the collagen solution and polymerizing. The cells were seeded.	- Cell viability - ALP - Immunofluorescence - Confocal microscopy - RTPCR - Alizarin red	- Enhanced proliferation and osteoblastic differentiation. - Higher early expression of ALP, COL I, OPN, and RUNX2 compared with 2D. - Formed mineralized nodules with calcium deposition by day 14.	3D collagen scaffolds demonstrated strong potential for promoting mineralized matrix formation in periodontal regeneration.
Yamada, et al. (2014) [35]	Dental epithelial cells + Periodontal fibroblasts (porcine)	Hydrated collagen gel	Collagen was mixed with DMEM and FBS. Cells were added.	- Immunohistochemical staining	- Formed stratified epithelium in vitro and in vivo. - In vitro, cytokeratin 8/18/19 and involucrin were expressed. - Only laminin and cytokeratin were detected in vivo.	3D cultures formed a stratified epithelial structure, suggesting that differentiation of three-dimensional culture tissues differs in vivo and in vitro.
Dabija-Wolter, et al. (2013) [54]	Gingival fibroblasts + Periodontal ligament fibroblasts (human)	Collagen matrix	Cell was seeded on top of a reconstituted collagen I biomatrix supplemented or not with fibroblasts.	- Immunohistochemical staining	- Reproduced JE and SE phenotypes depending on culture time and fibroblast type. - Day 5 cultures with PDLF resembled JE, day 7 cultures with GF or hPDLF resembled SE, and day 9 with GF resembled gingival epithelium. - Marker expression included ODAM, FDCSP, and CK19.	These models as reliable tools for studying periodontal bacteria–host interactions.
Lee, et al. (2007) [46]	Periodontal ligament cells (human)	Calf skin collagen gel	Cells were cultured in a three-dimensional collagen gel hydrogel.	- cDNA synthesis - RTPCR - ELISA - ALP	- Increased IL6 expression and decreased ALP activity, while IL8 showed no significant change.	The changed expression of IL-6 and ALP in response to the static compressive force in PDL cells suggests promotion of bone resorption and reduction in mineralization, supporting the role of PDL cells in orthodontic tooth movement.
Reuther, et al. (2003) [60]	Periodontal ligament fibroblasts + Alveolar bone cells (human)	Type I collagen matrix	Osteoblasts were incorporated into a collagen type I solution and polymerized. Pdl cells were seeded onto the osteoblast-containing collagen.	- Confocal microscopy - Gel contraction - Ki67 antigen detection	- Promoted sustained stratification of hPDLF cells, which was lost in monocultures. - Bone cells showed progressive stratification and strong gel contraction, likely driven by proliferation and migration. - Cocultures maintained proliferation, compartmentalization, and morphogenesis only in the 3D system.	A novel experimental tool to further elucidate the underlying mechanisms of the growth and differentiation of hPDLF and bone tissue.

**Table 4:** Summary of genes and proteins evaluated to determine cell differentiation.

Genes and Proteins	Number of Studies (*n*)	Differentiation	References
Activating Transcription Factor 4 (ATF4)	1	Cementogenic	[27]
Activating Transcription Factor 6 (ATF6)	1	Cementogenic	[27]
**Activating Transcription Factor 6 beta (ATF6β)**	1	Cementogenic	[27]
Alkaline Phosphatase	16	Osteogenic	[5,20,22,29,30,31,33,34,38,43,44,46,47,54,55,59]
**Alpha-Smooth Muscle Actin (α-SMA)**	1	Osteogenic	[20]
Ameloblastin (AMBN)	1	Chondrogenic/Epithelial	[9]
Amelotin (AMTN)	1	Chondrogenic/Epithelial	[9]
Amphiregulin (AREG)	1	Chondrogenic/Epithelial	[9]
Asporin (ASPN)	1	Cementogenic/Osteogenic	[5]
Autophagy-Related Gene 5 (ATG5)	1	Osteogenic/Adipogenic	[25]
**Bcl-2-Associated X Protein (BAX)**	1	Cementogenic	[27]
Beclin 1 (BECN1)	1	Osteogenic	[25]
Betacellulin (BTC)	1	Chondrogenic/Epithelial	[9]
Bone Gamma-Carboxyglutamate Protein (BGLAP)	1	Cementogenic/Osteogenic/Chondrogenic/Adipogenic/Epithelial	[28]
Bone Morphogenetic Protein 2 (BMP2)	2	Cementogenic/Osteogenic/Chondrogenic/Adipogenic/Epithelial	[28,32]
Bone Sialoprotein (BSP)	4	Cementogenic/Osteogenic	[40,44,49,57]
Calnexin (CANX)	1	Cementogenic/Adipogenic	[27]
Calreticulin (CALR)	1	Cementogenic/Adipogenic	[27]
Caspase 3 Apoptotic Cysteine Protease (CASP3)	1	Cementogenic/Adipogenic	[27]
Caspase 7 Apoptotic Cysteine Protease (CASP7)	1	Cementogenic/Adipogenic	[27]
Catabolite Activator Protein (CAP)	2	Cementogenic/Osteogenic	[44,54]
Cementum Protein 1 (CEMP1)	3	Cementogenic/Osteogenic	[24,54,57]
**Collagen Type I Alpha 1 Chain (COL1A1)**	13	Cementogenic/Osteogenic/Chondrogenic/Adipogenic/Epithelial	[5,24,28,32,34,38,40,42,46,49,53,57,59]
**Collagen Type II Alpha 1 Chain (COL2A1)**	1	Cementogenic/Osteogenic/Chondrogenic/Adipogenic/Epithelial	[28]
**Collagen Type III Alpha 1 Chain (COL3A1)**	5	Cementogenic/Osteogenic/Chondrogenic/Adipogenic	[24,27,28,53,59]
**Collagen Type XII Alpha 1 Chain (COL12A1)**	2	Cementogenic/Osteogenic	[24,49]
**Cyclic AMP-Responsive Element-Binding Protein 3 (CREB3)**	1	Cementogenic/Adipogenic	[27]
Cyclin D (CCND)	1	Epithelial	[60]
Cyclooxygenase-2 (COX2)	1	Cementogenic/Osteogenic	[40]
Cytokeratin (KRT)	1	Cementogenic/Osteogenic	[55]
Dentin Sialophosphoprotein (DSPP)	1	Cementogenic/Osteogenic	[57]
E-cadherin (CDH1)	1	Epithelial	[60]
Epidermal Growth Factor (EGF)	1	Chondrogenic/Epithelial	[9]
Epigen (EPGN)	1	Chondrogenic/Epithelial	[9]
Epiregulin (EREG)	1	Chondrogenic/Epithelial	[9]
Fibroblast Growth Factor 2 (FGF2)	2	Cementogenic/Osteogenic/Chondrogenic	[32,38]
GLI Family Zinc Finger 1 (GLI1)	1	Chondrogenic/Epithelial	[9]
**Heparin-Binding EGF-Like Growth Factor (HBEGF)**	1	Chondrogenic/Epithelial	[9]
Hepatocyte Growth Factor (HGF)	1	Cementogenic/Osteogenic	[24]
Integrin Subunit Alpha 6 (ITGA6)	1	Chondrogenic/Epithelial	[9]
Integrin-Linked Kinase (ILK)	1	Osteogenic	[25]
**Leucine-Rich Repeats and Immunoglobulin-Like Domains 1 (LRIG1)**	1	Chondrogenic/Ephitelial	[9]
Lysyl Oxidase (LOX)	1	Cementogenic/Adipogenic	[27]
**Matrix Metalloproteinase 1 and 3 (MMP1 and MMPIII)**	2	Cementogenic/Osteogenic/Chondrogenic/Adipogenic/Epithelial	[28]
Meis Homeobox 1 (MEIS1)	1	Chondrogenic/Epithelial	[9]
Myogenic Differentiation 1 (MYOD1)	1	Cementogenic/Osteogenic/Chondrogenic/Adipogenic/Epithelial	[28]
Nanog Homeobox (NANOG)	1	Chondrogenic/Epithelial	[30]
Neuregulin 1 (NRG1)	1	Chondrogenic/Epithelial	[9]
Neuregulin 2 (NRG2)	1	Chondrogenic/Epithelial	[9]
Neuregulin 3 (NRG3)	1	Chondrogenic/Epithelial	[9]
Neuregulin 4 (NRG4)	1	Chondrogenic/Epithelial	[9]
Octamer-binding Transcription 4 (Oct4)	1	Chondrogenic/Epithelial	[30]
**Odontogenic Ameloblast-Associated Protein (ODAM)**	2	Chondrogenic/Epithelial	[9,36]
Osteocalcin (OCN)	5	Cementogenic/Osteogenic	[24,33,34,49,53]
Osteopontin (OPN)	2	Cementogenic/Osteogenic	[33,34]
Osteoprotegerin (OPG)	1	Cementogenic/Osteogenic/Chondrogenic	[32]
Paired-Like Homeodomain 2 (PITX2)	1	Chondrogenic/Epithelial	[9]
**Periodontal Ligament-Associated Protein-1 (PLAP1)**	3	Cementogenic/Osteogenic/Chondrogenic	[24,32,38]
Periostin (POSTN)	5	Cementogenic/Osteogenic/Epithelial	[5,21,42,53,60]
Peroxisome Proliferator-Activated Receptor Gamma (PPARG)	1	Cementogenic/Osteogenic/Chondrogenic/Adipogenic/Epithelial	[28]
Phosphoinositide 3-Kinase (PI3K)	1	Osteogenic	[25]
**Receptor Activator of Nuclear Factor Kappa-**Β **Ligand (RANKL)**	1	Cementogenic/Osteogenic/Chondrogenic	[32]
**Runt-Related Transcription Factor 2 (Runx2)**	6	Cementogenic/Osteogenic/Chondrogenic/Adipogenic/Epithelial	[28,33,34,40,42,53]
**SRY-Box Transcription Factor 2 (Sox2)**	2	Chondrogenic/Epithelial	[9,30]
**SRY-Box Transcription Factor 21 (SOX21)**	1	Chondrogenic/Epithelial	[9]
**SRY-Box Transcription Factor 9 (SOX9)**	1	Cementogenic/Osteogenic/Chondrogenic/Adipogenic/Epithelial	[28]
Stem Cell Marker (STRO-1)	1	Cementogenic/Osteogenic	[56]
**T-Box Transcription Factor 1 (TBX1)**	1	Chondrogenic/Epithelial	[9]
**Tissue Inhibitor of Metalloproteinases 1 (TIMP1)**	1	Cementogenic/Osteogenic/Chondrogenic/Adipogenic/Epithelial	[28]
Transcription Factor Osterix (Sp7)	2	Cementogenic/Osteogenic/Chondrogenic/Adipogenic/Epithelial	[28,53]
**Transforming Growth Factor Beta 1 (TGFB1)**	1	Cementogenic/Osteogenic/Chondrogenic/Adipogenic/Epithelial	[28]
**Tumor Necrosis Factor Receptor Superfamily Member 11B (TNFRSF11B)**	1	Cementogenic/Osteogenic/Chondrogenic/Adipogenic/Epithelial	[28]
**Tumor Necrosis Factor Superfamily Member 11 (TNFSF11)**	1	Cementogenic/Osteogenic/Chondrogenic/Adipogenic/Epithelial	[28]
**Twist Family BHLH Transcription Factor 1 (TWIST1)**	1	Cementogenic/Osteogenic	[53]
**Vascular Endothelial Growth Factor A (VEGFA)**	1	Cementogenic/Osteogenic	[24]
Vimentin (VIM)	1	Cementogenic/Osteogenic	[56]
Yes-Associated Protein 1 (YaP1)	1	Cementogenic/Osteogenic	[53]
